# KVarPredDB: a database for predicting pathogenicity of missense sequence variants of keratin genes associated with genodermatoses

**DOI:** 10.1186/s40246-020-00295-z

**Published:** 2020-12-07

**Authors:** Yuyi Ying, Lu Lu, Santasree Banerjee, Lizhen Xu, Qiang Zhao, Hao Wu, Ruiqi Li, Xiao Xu, Hua Yu, Dante Neculai, Yongmei Xi, Fan Yang, Jiale Qin, Chen Li

**Affiliations:** 1 Department of Human Genetics, and Women’s Hospital, Zhejiang University School of Medicine, Hangzhou, China; 2 Zhejiang Provincial Key Laboratory of Genetic & Developmental Disorders, Zhejiang University School of Medicine, Hangzhou, China; 3 Department of Genetics, College of Basic Medical Sciences, Jilin University, Changchun, 130021 Jilin China; 4 Department of Basic Medical Sciences, Zhejiang University School of Medicine, Hangzhou, China; 5 Chu Kochen Honors College, Undergraduate School of Zhejiang University, Hangzhou, China; 6 Department of Ultrasound, Women’s Hospital, Zhejiang University School of Medicine, Hangzhou, China

**Keywords:** Keratin genes, Genodermatoses, Pathogenicity, Missense variants, Novel variants, Database

## Abstract

**Background:**

Germline variants of ten keratin genes (*K1*, *K2*, *K5*, *K6A*, *K6B*, *K9*, *K10*, *K14*, *K16*, and *K17*) have been reported for causing different types of genodermatoses with an autosomal dominant mode of inheritance. Among all the variants of these ten keratin genes, most of them are missense variants. Unlike pathogenic and likely pathogenic variants, understanding the clinical importance of novel missense variants or variants of uncertain significance (VUS) is the biggest challenge for clinicians or medical geneticists. Functional characterization is the only way to understand the clinical association of novel missense variants or VUS but it is time consuming, costly, and depends on the availability of patient’s samples. Existing databases report the pathogenic variants of the keratin genes, but never emphasize the systematic effects of these variants on keratin protein structure and genotype-phenotype correlation.

**Results:**

To address this need, we developed a comprehensive database KVarPredDB, which contains information of all ten keratin genes associated with genodermatoses. We integrated and curated 400 reported pathogenic missense variants as well as 4629 missense VUS. KVarPredDB predicts the pathogenicity of novel missense variants as well as to understand the severity of disease phenotype, based on four criteria; firstly, the difference in physico-chemical properties between the wild type and substituted amino acids; secondly, the loss of inter/intra-chain interactions; thirdly, evolutionary conservation of the wild type amino acids and lastly, the effect of the substituted amino acids in the heptad repeat. Molecular docking simulations based on resolved crystal structures were adopted to predict stability changes and get the binding energy to compare the wild type protein with the mutated one. We use this basic information to determine the structural and functional impact of novel missense variants on the keratin coiled-coil heterodimer. KVarPredDB was built under the integrative web application development framework SSM (SpringBoot, Spring MVC, MyBatis) and implemented in Java, Bootstrap, React-mutation-mapper, MySQL, Tomcat. The website can be accessed through http://bioinfo.zju.edu.cn/KVarPredDB. The genomic variants and analysis results are freely available under the Creative Commons license.

**Conclusions:**

KVarPredDB provides an intuitive and user-friendly interface with computational analytical investigation for each missense variant of the keratin genes associated with genodermatoses.

## Background

Human epidermis is the outermost layer of the skin, which plays a protective role in human body. The epidermis is majorly composed of keratinocytes (> 90%) [1]. To achieve the environment-barrier function, it should be intact and undisturbed with an integrated cytoskeletal network [2]. The cytoskeleton majorly consists of three groups of filaments: intermediate filaments (IFs), microfilaments (MFs), and microtubules (MTs) [3]. Based on the requirements for different activities, usage, and developmental stages, the structure of cytoskeleton varies from rigid to flexible. Recent research showed that the mechanical properties of keratinocytes are largely affected by the capability of the IFs [4], especially keratins [1], which are the most abundant structural proteins in keratinocytes [5].

Keratin (K), which is one of the largest gene families with 54 genes, is divided between type I (*K9-K40*/acidic) and type II (*K1-K8*, *K71-K86*/neutral or basic) keratins [6]. Type I and type II keratin usually bind together to form a coiled-coil heterodimer, the unit of the keratin cytoskeleton. Germline variants of ten keratin genes (*K1*, *K2*, *K5*, *K6A*, *K6B*, *K9*, *K10*, *K14*, *K16*, and *K17*) exert dominant-negative effect in the formation of keratin coiled-coil heterodimer, which finally leads to a large group of clinically and genetically heterogeneous genodermatoses [1, 7, 8]. Keratin genes associated genodermatoses usually manifested with keratinocyte fragility, blistering, and thickening of the palmoplantar epidermis [9]. Location of the missense mutation is also a significant factor for understanding the clinical severity of keratin-related genodermatoses. Keratin missense variants located in the helix initiation motif of 1A domain, termination motif of 2B domain and the L12 linker domain  have been reported for causing more severe clinical manifestations. Hundreds of mutations in these ten keratin genes have been reported for causing many types of genodermatoses [10].

Clinical significance of previously reported pathogenic variants as well as likely pathogenic (majorly *loss-of-function*) variants are very easy to understand because *loss-of-function* mutations always cause loss of protein product. Meanwhile, understanding the clinical importance of novel missense variants or variants of uncertain significance (VUS) is the biggest challenge due to the difficulty of interpreting whether it is associated with disease or not without doing structural or functional analysis [11]. Note that as recommended in Standards and Guidelines for the Interpretation of Sequence Variants: A Joint Consensus Recommendation of the ACMG [12], the terms “mutation” and “polymorphism” are recommended to be replaced by the term “variant” with the following modifiers: (1) pathogenic, (2) likely pathogenic, (3) uncertain significance, (4) likely benign, or (5) benign. In this paper, we thus practice these updated standards and guidelines for classification of sequence variants.

Next-generation sequencing technologies were performed to identify the candidate gene with their disease-causing variants in patients with genodermatoses. During data analysis and variant interpretation, we usually identified many novel missense variants with low minor allele frequency (MAF), but we were unable to understand their pathogenicity and possible potential for causing genodermatoses. Functional characterization is the only way to understand the clinical association of those novel missense variants or VUS but it is time consuming, costly, and depends on the availability of patient’s samples. At present, none of the database or web servers assessed and predicted the potential pathogenicity of keratin missense variants, i.e., their effect on keratin protein structure and genotype-phenotype correlation.

In addition, target-based therapeutic or precision medicine to cure these genodermatoses is remaining a great challenge [13]. Hence, due to incurability of keratin-related genodermatoses, prenatal diagnosis is the best way to prevent the occurrence of such inherited diseases. A comprehensive database focusing on the human genodermatoses as well as predicting pathogenicity for novel missense variants of uncertain or unknown significance allows geneticists to evaluate the effect of the variants. It finally enables the clinicians for timely and accurate diagnosis, management, and follow-up.

So far, the most comprehensive database for keratins is the Human Intermediate filament Database (Interfil) [14]. It provides general information of intermediate filament encoded genes including mRNA sequence, protein sequence, domain information, and germline mutations associated with disease in a tabular collection format. Unfortunately, no updates have appeared since March 2017. There are also several commonly used databases providing the information of all the reported missense variants of keratin genes, such as dbSNP [15], The Human Gene Mutation Database (HGMD) [16, 17], and LOVD [18]. None of these databases provides any information regarding the effect of missense variants on keratin structure and genotype-phenotype correlation, which is highly important for investigating the genetic effect and the epigenetic interference behind the phenotype. In order to aid users to quest a missense variant of keratin gene to understand the molecular basis of genodermatoses and its genotype-phenotype correlation, we present a more comprehensive and detailed online database for human genodermatoses related keratin genes with an intuitive and user-friendly interface, namely, KVarPredDB.

We visualized manually curated missense sequence variants of all ten keratin genes, diseases, and the analyses of changes in physico-chemical characteristics between wild types and substituted amino acids as well as protein structural effect on the keratin coiled-coil heterodimer complex for each missense variant. The resulting predicted tendencies could be validated by in vivo/vitro experiments and provides guidance to genetic counselors and clinicians for accurate prenatal diagnosis.

## Results

### Data coverage and statistics

KVarPredDB contains missense variant information of ten keratin genes (*K1*, *K2*, *K5*, *K6A*, *K6B*, *K9*, *K10*, *K14*, *K9*, *K16*, and *K17*) reported to be associated with genodermatoses, including 400 reported pathogenic missense variants and 4629 missense VUS. Table 1 showed the number of missense sequence variants in ten keratin genes, respectively.

**Table 1:** Overview of data represented in KVarPredDB

Keratin gene	HGNC ID	Pathogenic variants	Variants of uncertain significance (VUS)	Total
*K1*	HGNC:6412	41	489	530
*K10*	HGNC:6413	35	429	464
*K5*	HGNC:6442	117	448	565
*K14*	HGNC:6416	82	348	430
*K2*	HGNC:6439	15	480	495
*K9*	HGNC:6447	27	535	562
*K6A*	HGNC:6443	35	508	543
*K6B*	HGNC:6444	4	598	602
*K16*	HGNC:6423	19	412	431
*K17*	HGNC:6427	25	382	407
Total		400	4629	5029

Except for sorting out 27 diseases and ethnic information of the patient harboring pathogenic missense variants, we also analyze the changes in the physico-chemical characteristics, inter/intra-chain interatomic interaction, evolutionary conservation, and heptad repeat location on the stability and assembly competence of the mutated keratin coiled-coil heterodimer based on our previous studies [19, 20]. We complete the molecular docking simulation of three protein structures, i.e., K1/K10-2B (4ZRY) [5], K5/K14-2B (3TNU) [21], and K1/K10-1B (6EC0) [22] and 332 missense sequence variants on them.

### Querying the database

To facilitate the data interpretation, graphical representations with interactive features were developed. Querying the database is primarily based on either “cDNA Variant” or “Disease” under the “Search” menu. From the “cDNA Variant” page, users can quickly access the database by choosing “Gene Symbol,” “Pathogenic Missense Variants,” or “Uncertain Significance Missense Variants” which are implemented by a drop-down list box. Here, the term “Uncertain Significance Missense Variants” refers to missense sequence variants of the VUS, while “Pathogenic Missense Variants” indicates reported missense sequence variants reported to cause genodermatoses. For each missense variant entry, a lollipop plot, gene symbol, protein structural analysis (including changes in physico-chemical properties, inter/intra-chain interaction, evolutionary conservation, location in heptad repeats analyses), patient’s ethnicity/population information, related disease, molecular-docking result, and related references are displayed by default. On the “Disease” page under the “Search” menu, users are asked to select one of 27 types of genodermatoses curated in our database, which leads to the result page showing disease descriptions and missense variations in different keratin genes that cause the corresponding disease by lollipop plot together with related information on the variation in a tabular collection format. If different keratin genes cause the disease, these genes can be switched on the lollipop plot.

Under the “Browse” menu, the user can navigate “Keratin Genes/Proteins,” “Disease List,” and “Protein Structure & Molecular Docking.” “Keratin Genes/Proteins” provides a comprehensive overview of selected keratin genes/proteins in terms of protein description, coding sequence for downloading, missense variations distributed on the linearized keratin protein and its domains by lollipop plot, and lists of pathogenic missense variants and VUS. “Disease List” leads to collection of 27 types of genodermatoses, with respective OMIM ID, which is clickable and will take users to the corresponding disease page. A three-dimensional presentation of the determined crystal structure (1B and 2B domains of K1/K10, and 2B domain of K5/K14 heterodimers) is displayed on the “Protein structure & Molecular Docking” page, powered by NGL Viewer for 3D structure viewing. Lower panel on the same page, the “Molecular Docking Results” section shows all keratin gene missense sequence variants’ binding energy of wild type and the lowest binding energy score of mutant models.

The “API” menu contains all the application-programming interfaces. The application API provides a way to programmatically access our data including molecular docking information for each variant, related missense variants for each disease, and so on. Users, especially researchers, can provide novel keratin missense sequence variants to us by the “Submit” menu. We will verify and update the missense sequence variant information.

Instructions on how to query KVarPredDB are provided on the “Tutorials” page linked from the home page. At the same time, the “About” menu also contains KVarPredDB introduction information. The KVarPredDB runs in most common web browsers. Every element like number, image, or plot in the database is interactive with clickable buttons, and is thus far more user friendly and provides more easily accessible information.

## Discussion

KVarPredDB mainly works on integration and the prediction of pathogenicity of germline missense sequence variants of ten keratin genes associated with genodermatoses. As we noted above, keratin is one of the largest multi-gene families encoding structural proteins, with extremely high tissue-specific expression. Keratin-related genodermatoses are rare, incurable, and with an autosomal dominant mode of inheritance. Existing databases have reported the pathogenic missense variants of these genes, but have never emphasize on the systematic effect of these missense mutations on keratin protein structure and genotype-phenotype correlation. This hampers the investigation of the genetic effect and the epigenetic interference behind the phenotype.

Within this database, we visualized manually curated keratin gene missense sequence variants, diseases, patients’ ethnicity, and the analyses of changes in physico-chemical characteristics between the wild and mutant amino acids as well as protein structural effects on the keratin coiled-coil heterodimer for each missense variant. KVarPredDB also provides computational analysis results for users to assess the potential pathogenicity of missense VUS, based on a more comprehensive systematic analyses than common pathogenicity prediction tools such as SIFT [23], Polyphen-2 [24], DUET [25], and MutationAssessor [26, 27]. The resulting pathogenic tendencies could be further validated by in vivo/vitro experiments and provide guidance to genetic counselors and clinicians for easy and accurate prenatal diagnosis. These in silico tools aid in the interpretation of missense sequence variants. The algorithms used by each tool may differ from each other but could be divided into two main categories: one predicts whether the variant is damaging to the resultant protein function or structure, and the other predicts the effect on splicing at the nucleotide level. None of these tools is sufficient to reveal the possible mechanism behind the phenotype from both physic-chemical and structural points of view. Searching in our database, every user can easily get the information related to pathogenicity of missense sequence variants of keratin genes. It will give keratin-related-disease families a chance for getting a healthy baby, and provide new ideas for genetic counseling. It will be a valuable way for minimizing the risk of occurrence of keratin-related genodermatoses.

At present, only three partial crystal structures of keratin proteins have been published. We will continue to finish the work with K1/K10-1B domain (6EC0) [22]. With the aids of crystallization and cyto-electron microscopy (Cryo-EM) techniques, a high resolution of the class of intermediate filament proteins will allow us to perform more accurate predictions. For the protein domains without crystal structure, analysis will be conducted via homology modeling owing to their high structural similarities. Next, we will continue to update the missense sequence variants and related information to ensure the timeliness of the database.

## Conclusions

KVarPredDB provides the computational analytical investigation for each missense variant of the keratin genes, which could be further validated by in vivo/vitro experiments or provide guidance to genetic counselors and clinicians for easy and accurate prenatal diagnosis. Meanwhile, we will investigate the protein structural and assembly characteristics of more types of coding variations to further improve our analysis strategies for the pathogenicity prediction.

## Methods

### Data acquisition

**Fig. 1:**
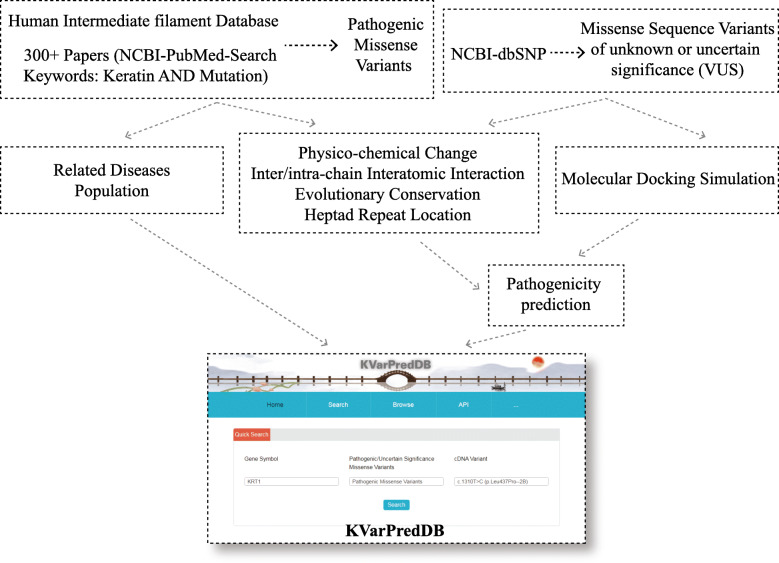
Schematic presentation of data integration and curation of KVarPredDB

KVarPredDB contains information of ten keratin genes reported to be related to genodermatoses. All this information was integrated and manually curated from the Interfil, NCBI-dbSNP, NCBI-PubMed (before 30 October 2020) (Fig. 1). In particular, pathogenic missense variants were extracted and integrated from the Interfil. Related references in the NCBI-PubMed were searched with the keywords “mutation” and “keratin” dated from March 2017. Meanwhile, VUS were mainly extracted and curated from NCBI-dbSNP. All this variant information is presented in two ways that are easily accessible (i.e., text and lollipop diagram to the users [28]).

We also integrated and enlisted all types of keratin gene associated diseases with a hyperlink to the Online Mendelian Inheritance in Man (OMIM) and classified these pathogenic missense variants according to different disease types. KVarPredDB displayed the complete keratin information in the database, including gene and protein information, such as keratin-coding sequence obtained from Ensembl and Uniprot. Also, KVarPredDB had all the ethnic or population information of the patient identified with the reported pathogenic missense variants.

Due to the particularly long fibrous structure of keratin, only part of the crystal structures was available for protein structure analyses, i.e., K1/K10-2B domain (4ZRY) [5], K5/K14-2B domain (3TNU) [21], and K1/K10-1B domain (6EC0) [22], which was retrieved from RCSB Protein Data Bank.

### Pathogenicity prediction

Analyses of pathogenicity for each missense variant were performed according to two parts, i.e., our previous computational studies [19, 20] and molecular docking methods based on resolved crystal structures.

**Fig. 2:**
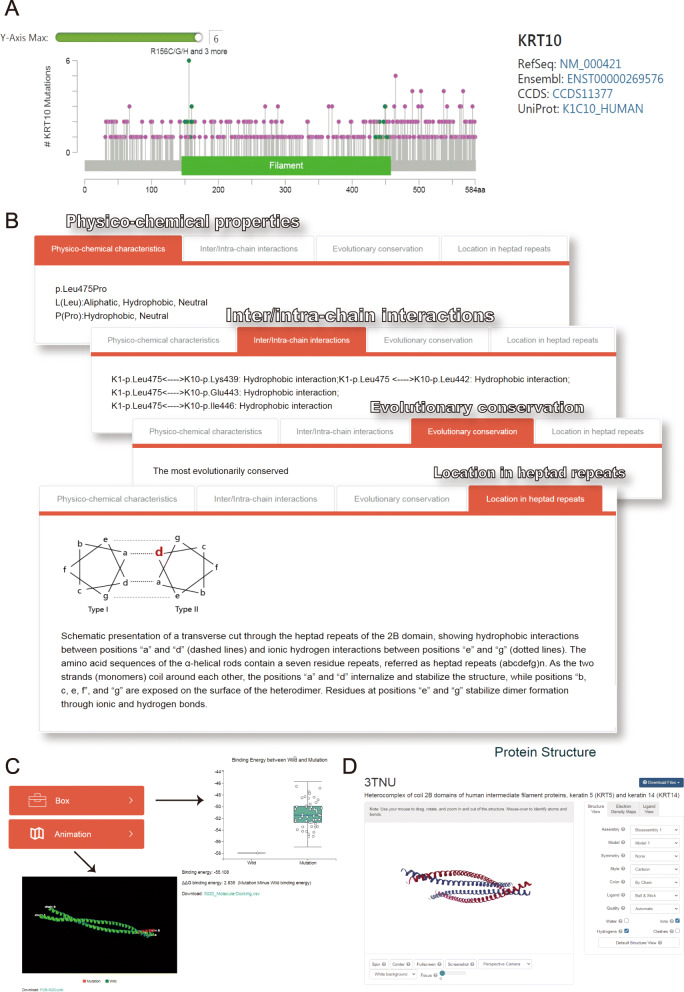
Example outputs of KVarPredDB. **a** A lollipop diagram is displayed to show the distribution of all the missense sequence variations on a linearized *K10* protein and its domains. *Y*-axis represents *K10* sequence variant frequency. **b** Structural analyses in terms of physico-chemical properties, inter/intra-chain interactions, evolutionary conservation, and location in heptad repeats. **c** Visualization of ddg score generated by molecular docking simulation (box plot) and structural alignment (GIF format) between the wild type and mutant keratin coiled-coil heterodimer. **d** Screenshot of K5/K14 heterodimer coiled-coil complex structure (3TNU) in the “NGL Viewer” GUI

KVarPredDB is providing the detailed information including changes in physico-chemical characteristics, inter/intra-chain interaction, evolutionary conservation, and heptad repeat location to understand the stability and assembly competence of the keratin coiled-coil heterodimer upon missense variants (Fig. 2). We can use this basic information to determine the structural and functional impact of variants on the keratin coiled-coil heterodimer.

Besides, molecular docking simulations were adopted to predict stability changes and get the binding energy to compare the wild type protein with the mutated one. The structure was first relaxed using the Rosetta relax application [29]. We used the relax application in order to find the most energetically favorable conformation of the protein. The Monte Carlo (MC) algorithm generates conformational structure changes; the energy of the new conformation is calculated and compared with the energy before change. If the energy is better, the change is accepted. We run 50 repeats of the relax application and choose the best one as input. We took the relaxed rada recombinase as the wild type and then used Rosetta backrub application to generate a structural model of the mutant. Rosetta Backrub application attempts to capture the tiny conformational changes with the protein. The protein backrub is first divided into multiple fragments; each of the fragments rotates around the connecting axis while immobilizing the rest of the protein. Rotational movements have six internal backbone degrees of freedom: φ, ψ, and N-Cα-C bond angle at each pivot. The side chain repackaging and energy minimization follow along with all torsion angles. We repeated this process for 1000 trials and selected the lowest energy structure. The resulting conformations were scored with the Rosetta scoring function and accepted (or rejected) according to the Metropolis criterion using a kT of 0.6. For each missense variant, at least 50 models were generated. To predict the change in stability of rada recombinase mutant protein induces by a missense variant, models are further screened with the ddG method in Rosetta Script, chain number is 2. DdG refers to binding energy and gives the differences in Rosetta energy between the wild and mutant protein structure. Rosetta energy function Talaris2014 was used. Finally, we visualize the difference between wild and mutant protein structure with box plot chart and structural alignment.

### Database construction

A user-friendly web interface was developed with Java 1.8. All data was implemented with MySQL (version 5.6). The tables included diseases, pathogenic/uncertain significance missense variants, references, proteins, amino acid physico-chemical properties, and molecular mocking results. The back end used a three-tier model: customer display layer, business logic layer, and data layer, which has good flexibility, scalability, and shareability. It was built under SpringBoot (version 2.1.6), Spring MVC, and Mybatis (version 2.0.1) framework. The front end was implemented using Bootstrap (version 3.3.7) and jQuery. The pages used Ajax, which refers to a web development technology for creating interactive, fast and dynamic web applications. It can update web pages without reloading the entire web page.

A lollipop plot (also known as stick or needle plots) generated by the MutationMapper visualization tool [28, 30] displays the distribution of all missense variants of a linearized keratin protein and its domains (genome build GRCh37/hg19). An embedded module from RCSC supported by NGL viewer (ngl.js) was employed to display molecular graphics [31]. Embed Tomcat was used for the server. The website design was responsive. The database is routinely updated and, as such, the quantity of analysis and accuracy will continue to increase as more case reports and determined crystal structures are added.

### Accession numbers

Atomic coordinates and structure factors for the reported crystal structures are retrieved from the Protein Data bank under accession number 3TNU, 4ZRY, and 6EC0.

Searching for keratin gene variants with NCBI-dbSNP under accession number 3848, 3849, 3852, 3853, 3854, 3857, 3858, 3861, 3868, and 3872.

## Data Availability

The data that support the findings of this study are available from the corresponding author upon reasonable request.
